# Enuresis in children and adolescents with sickle cell anaemia is more frequent and substantially different from the general population

**DOI:** 10.1371/journal.pone.0201860

**Published:** 2018-08-10

**Authors:** Christopher Imokhuede Esezobor, Patricia Akintan, Uche Nwaogazie, Edna Akinwunmi, Edamisan Temiye, Adebola Akinsulie, Rasheed Gbadegesin

**Affiliations:** 1 Department of Paediatrics, Faculty of Clinical Sciences, College of Medicine University of Lagos, Lagos, Nigeria; 2 Department of Paediatrics, Lagos University Teaching Hospital, Mushin, Lagos, Nigeria; 3 Department of Pediatrics, Division of Nephrology, Duke University Medical Center, Durham, NC, United States of America; 4 Duke Molecular Physiology Institute, Durham, NC, United States of America; University of Alabama at Birmingham, UNITED STATES

## Abstract

**Background:**

No large studies have examined the prevalence of enuresis, its various forms and risk factors in children with sickle cell anaemia (SCA) in Sub-Saharan Africa using standardised definitions. We determined age and gender-specific prevalence of enuresis and compared the nature of enuresis in children with and without SCA. We also identified predictors of enuresis in children with SCA.

**Methods:**

Caregivers of children with SCA attending a tertiary centre haematology clinic in Nigeria were interviewed using a questionnaire. In addition, a separate questionnaire was completed for every sibling aged 5–17 years whose haemoglobin genotype was known. Enuresis and its various forms were defined using the definitions of the International Children’s Continence Society.

**Results:**

The study involved 243 children with SCA and 243 controls matched for age and sex. The mean age of the study cohort was 9.9 (3.4). Females made up 45.7% of the cohorts. The prevalence of enuresis was 49.4% and 29.6% in children with and without SCA, respectively (p = 0.009). In both groups, the prevalence of enuresis declined with age but remained five times higher at 25% in children with SCA aged 14–17 years compared with controls. Also, children with SCA and enuresis were older, more likely to have non-monosymptomatic enuresis and wet at least three nights per week than controls. Independent predictors of enuresis in children with SCA were a family history of enuresis and young age.

**Conclusion:**

Children with SCA had more frequent and more severe enuresis which persisted to late adolescence than age and sex-matched controls. These features indicate a subset of enuresis that is difficult to treat in the general population. Young age and enuresis in a family member define a subset of children with SCA more likely to have enuresis. Healthcare workers need to discuss enuresis with parents of children with SCA and offer referral to continence services.

## Introduction

Enuresis is common in children and adolescents with sickle cell anaemia (SCA), with prevalence of 20 to 58% [1, 2]. In most cases, the rates are about two folds higher than in the general populations [3, 4]. Several explanations have been put forward to explain the strong association between enuresis and SCA. Notable among these explanations are polyuria from hyposthenuria, sleep disordered breathing and functionally small bladder [5–7]. Sub-Saharan Africa has the highest burden of SCA with over 230,000 births with SCA each year [8]. Paradoxically, most large studies describing enuresis in SCA have been largely performed in developed regions of the world where the severity of SCA is relatively milder and the spectrum of disease-ameliorating health interventions more comprehensive than in Sub-Saharan Africa [6, 9]. The few studies investigating sickle cell disease and enuresis in Sub-Saharan Africa were either underpowered to answer questions such as age-specific prevalence of enuresis or used non-standardised definitions which makes comparisons with other studies difficult [3, 10]. Furthermore, in these studies no clear distinction was made between the various forms of enuresis, such as secondary and non-monosymptomatic enuresis, which requires different approaches to management [4, 10, 11].

We hypothesised that the epidemiology of enuresis in children and adolescents with SCA in sub-Saharan Africa would be substantially different from that described for children in developed regions of the world. In the general population, childhood enuresis is more common in developing regions of the world than in developed regions, implying some role for socioeconomic factors in the occurrence or resolution of enuresis. In the present study, we determined the prevalence of enuresis and its various forms in children and adolescents with SCA and compared them with age and sex-matched controls. Furthermore, we determined the age and gender-specific rates of enuresis and identified independent predictors of enuresis in children and adolescents with SCA.

## Materials and methods

Between 2015 and 2018, we interviewed primary caregivers of children with SCA aged 5 to 17 years attending the Paediatric Haematology Clinic of a public tertiary hospital in Lagos. The interview was conducted using a structured, pretested questionnaire (S1 Survey Instrument). In addition, a separate questionnaire was completed for every sibling of children with SCA aged 5 to 17 years whose haemoglobin genotype was known. To ensure a 1:1 matching in terms of age and sex with the children with SCA, 94 children from a database of over 900 children aged 5 to 17 years were added to 149 siblings of children with SCA in the control group. The database was derived from a community-based study on enuresis conducted in 2014 by one of the investigators of the present study [12]. To minimise bias, the selection from the database was done after blinding of the enuresis status.

### Data collection tool

The questionnaire has previously been described [12]. In summary, the questionnaire had sections on the demographics of the child and family, presence, severity and types of enuresis, presence of lower urinary tract symptoms such as urgency, history of bedwetting in siblings or parents and the level of caregiver concern about enuresis in the child using a scale of 1 to 10, with 1 and 10 meaning least concerned and greatly concerned, respectively. A new modification to the questionnaire was the inclusion of a separate section featuring questions pertaining to SCA, such as the age at diagnosis of SCA, number of hospitalisations in the 12 months prior to the study and history of blood transfusion.

### Ethics consideration

The study protocol was approved by the Health Research Ethics Committee of the Lagos University Teaching Hospital and conducted in accordance with the principles expressed in the Declaration of Helsinki. Also, the primary caregivers of children included in the study provided written informed consent before participating in the study.

### Definition of terms

Except otherwise specified the standardised definitions recommended by the International Children’s Continence Society were used in the study [13]. In summary, enuresis was defined as the presence of intermittent urinary incontinence during sleep at least once per month persisting for at least three months in children five years or older. Primary enuresis was defined as enuresis without dry period of at least six consecutive months. We defined non-monosymptomatic enuresis as the presence of urgency indicated as ‘always running to void’ or ‘cannot hold urine for a while even when told to do so’. The socioeconomic status of the family was classified using the method described by Olusanya et al [14] which uses the highest educational achievement of the mother and the father’s occupation.

### Data analysis

The data was analysed using IBM SPSS Statistics version 21 (IBM Corporation 2012, USA). Categorical variables were presented as proportions while normally distributed variables such as the age of the child were summarised as mean with standard deviation. Comparison of normally distributed data between two groups was done using Student’s t-test while the chi-squared test was used for proportions. We performed four sequential comparisons. First, we compared children with SCA with age and sex matched controls to determine if enuresis was more common in children with SCA than in the controls. Next, we compared children with SCA and enuresis and controls with enuresis to identify differences in the nature of enuresis between the two groups. Third, we compared children with SCA with and without enuresis in order to identify factors associated with enuresis in children with SCA. Last, we performed logistic regression analysis to identify independent predictors of enuresis in children with SCA. In the model, the dependent variable was the presence of enuresis and the covariates included factors reported in the literature to be associated with enuresis in children with SCA. The level of significance was set at a p value less than 0.05.

## Results

### Characteristics of the study population

We enrolled 243 children with SCA and 243 children without SCA. The mean age of the study cohort was 9.9 (3.4) years with 45.3% at least 10 years. Females made up 45.7% of the study population. Both groups were similar in terms of age, gender distribution, proportion whose parents were married and belonging to a family with a history of enuresis in siblings or parents. However, more children with SCA were firstborn, belonged to families with fewer children and higher socioeconomic status than controls (Table 1).

**Table 1:** Demographics of the study participants.

Characteristics	All children n = 486	Children with SCA n = 243	Controls n = 243	P value
Age, mean (SD), year	9.9 (3.4)	9.9 (3.5)	9.9 (3.4)	0.860
Adolescents (age ≥10 years), n, (%)	220 (45.3)	110 (45.3)	110 (45.3)	1.000
Female, n (%)	222 (45.7)	111 (45.7)	111 (45.7)	1.000
Number of children per family 1 2 3 4 ≥5	17 (3.5) 106 (21.8) 177 (36.4) 127 (26.1) 59 (12.1)	16 (6.6) 65 (26.7) 86 (35.4) 49 (20.2) 27 (11.1)	1 (0.4) 41 (16.9) 91 (37.4) 78 (32.1) 32 (13.2)	0.000
Birth order of child, n (%) First Second Third Fourth or higher	171 (35.2) 153 (31.5) 103 (21.2) 59 (12.1)	100 (41.2) 68 (28.0) 42 (17.3) 33 (13.6)	71 (29.2) 85 (35.0) 61 (25.1) 26 (10.7)	0.011
Socioeconomic class, n (%) Low Mid High	250 (51.4) 192 (39.5) 44 (9.1)	105 (43.2) 112 (46.1) 26 (10.7)	145 (59.7) 80 (32.9) 18 (7.4)	0.001
Married parents, n (%)	425 (87.4)	211 (86.8)	214 (88.1)	0.681
Family history of childhood enuresis, n (%)	233 (45.9)	108 (44.4)	115 (47.3)	0.524

### Age and sex specific prevalence of enuresis

**Fig 1:**
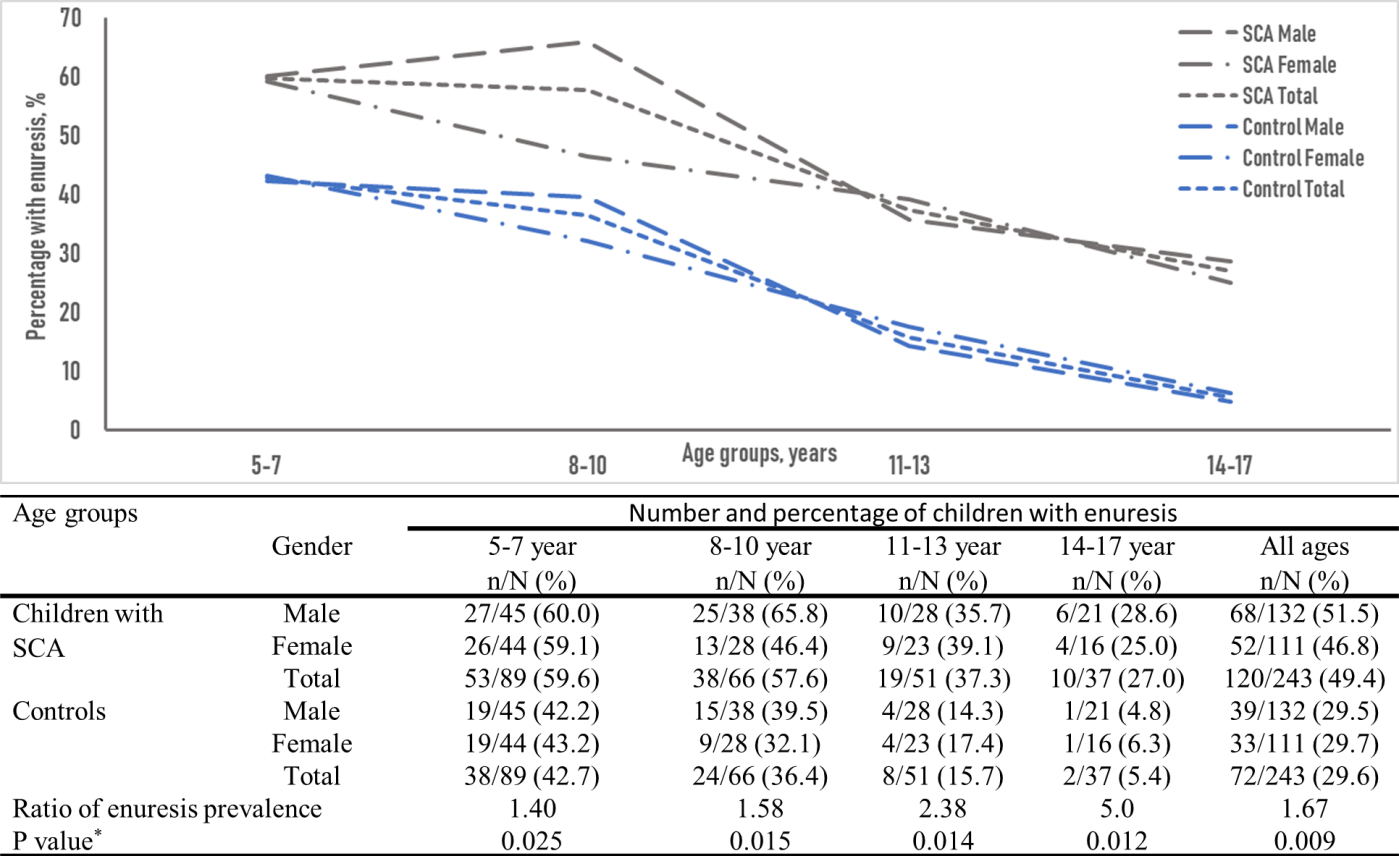
Age and sex-related prevalence of enuresis. *The p value compares the prevalence of enuresis in the group with sickle cell anaemia with the control.

The overall prevalence of enuresis was 49.4% in children with SCA and 29.6% in the controls (p = 0.009). With increasing age, there was a noticeable decline in the prevalence of enuresis in both groups of children. For children with SCA, enuresis rate declined from 59.6% in those aged 5 to 7 years to 27% in those 14 to17 years. Similarly, among the controls, the rate declined from 42.7% to 5.4%. However, ratio of the enuresis prevalence in both groups widened with increasing age, from a ratio of 1.40 in those 5 to 7 years to 5.0 in those aged 14 to 17 years (Fig 1).

The prevalence of enuresis varied using different wetting frequency (Table 2). Using the other commonly used definition of bedwetting of at least twice per week, 33.3% and 12.3% of the children with SCA and controls, respectively, had enuresis. Furthermore, children with SCA and enuresis had more frequent wet nights than controls with enuresis (p = 0.030).

**Table 2:** Severity and frequency of enuresis in children with and without sickle cell anaemia.

Wet nights per week	Prevalence of enuresis by frequency of wets*		Frequency of wets ^#^	
	Children with SCA n = 243 (%)	Controls n = 243 (%)	Children with SCA n = 120 (%)	Controls n = 72 (%)
Every day of the week	25 (10.3)	5 (2.1)	25 (20.8)	5 (6.9)
Six times per week	27 (11.1)	6 (2.5)	2 (1.7)	1 (1.4)
Five times per week	29 (11.9)	8 (3.3)	2 (1.7)	2 (2.8)
Four times per week	35 (14.4)	12 (4.9)	6 (5.0)	4 (5.6)
Thrice per week	54 (22.2)	20 (8.2)	19 (15.8)	8 (11.1)
Twice per week	81 (33.3)	30 (12.3)	27 (22.5)	10 (13.9)
Once per week	97 (39.9)	46 (18.9)	16 (13.)	16 (22.2)
Once per month	120 (49.4)	72 (29.6)	23 (19.2)	26 (36.1)
At least thrice per week	54 (22.2)	20 (8.2)	54 (45.0)	20 (27.8)

*P = 0.009

^#^P = 0.030

### Characteristics of enuresis in children with sickle cell anaemia

Children with SCA and enuresis were older (9.0 versus 8.2 years), more likely to be adolescents (34.2 versus 16.7%) and belonged to families with fewer children than controls with enuresis. These differences existed despite similar socioeconomic status and family history of enuresis. In addition, enuresis in children with SCA was twice more likely to be non-monosymptomatic (59.2 versus 29.2%) than in the controls. However, secondary enuresis occurred with similar frequency in both groups (10.0 and 16.7%) (Table 3).

**Table 3:** Comparing enuresis in children with sickle cell anaemia and age and sex matched controls.

Study participants’ characteristics	All children with enuresis n = 192	Children with enuresis		P value
		SCA, n = 120	Control, n = 72	
Age, mean (SD), year	8.7 (2.8)	9.0 (3.1)	8.2 (2.4)	0.041
Adolescents (age ≥10 years), n, (%)	53 (27.6)	41 (34.2)	12 (16.7)	0.009
Female, n (%)	85 (44.3)	52 (43.3)	33 (45.8)	0.736
Number of children per family 1 2 3 4 ≥5	5 (2.6) 41 (21.4) 73 (38.0) 50 (26.0) 23 (12.0)	5 (4.2) 36 (30.0) 40 (33.3) 27 (22.5) 12 (10.0)	0 (0.0) 5 (6.9) 33 (45.8) 23 (31.9) 11 (15.3)	0.001
Birth order of child, n (%) First Second Third Fourth or higher	62 (32.3) 66 (34.4) 43 (22.4) 21 (10.9)	47 (39.2) 37 (30.8) 23 (19.2) 13 (10.8)	15 (20.8) 29 (40.3) 20 (27.8) 8 (11.1)	0.062
Socioeconomic class, n (%) Low Mid High	103 (53.6) 76 (39.6) 13 (6.8)	61 (50.8) 49 (40.8) 10 (8.3)	42 (58.3) 27 (37.5) 3 (4.2)	0.416
Married parents, n (%)	164 (85.4)	101 (84.2)	63 (87.5)	0.526
Family history of childhood enuresis, n (%)	109 (56.8)	65 (54.2)	44 (61.1)	0.347
Non-monosymptomatic enuresis, n (%)	92 (47.9)	71 (59.2)	21 (29.2)	0.000
Secondary enuresis, n (%)	24 (12.5)	12 (10.0)	12 (16.7)	0.176

Only 13 (10.8%) primary caregivers of children with SCA and enuresis and three (4.2%) caregivers of controls with enuresis had spoken to a medical doctor about enuresis in their children (p = 0.106) despite level of concern greater than 5/10 in 42% and 34.7% of parents of those with SCA and controls, respectively.

### Factors associated with enuresis in children with sickle cell anaemia

Among children with SCA, those with enuresis were younger (9.0 versus 10.8 years) and had a family member with childhood enuresis (54.2 versus 35.0%). Although, more children with SCA and enuresis belonged to families with low socioeconomic class, the difference was not statistically significant. In contrast, gender, birth order, and number of children in the family were not associated with the presence of enuresis in children with SCA (Table 4)

**Table 4:** Factors associated with enuresis in children with sickle cell anaemia.

Study participants’ characteristics	All children with SCA n = 243	Children with SCA		P value
		Enuresis n = 120	No enuresis n = 123	
Mean age (SD), year	9.9 (3.5)	9.0 (3.1)	10.8 (3.6)	0.000
Adolescents (age ≥10 years), n, (%)	110 (45.3)	41 (34.2)	69 (56.1)	0.001
Female gender, n (%)	111 (45.7)	52 (43.3)	59 (48.0)	0.468
Socioeconomic class, n (%) Low Mid High	105 (43.2) 112 (46.1) 26 (10.7)	61 (50.8) 49 (40.8) 10 (8.3)	44 (35.8) 63 (51.2) 16 (13.0)	0.054
Number of children per family, n, (%) 1 2 3 4 ≥5	16 (6.6) 65 (26.7) 86 (35.4) 49 (20.2) 27 (11.1)	5 (4.2) 36 (30.0) 40 (33.3) 27 (22.5) 12 (10.0)	11 (8.9) 29 (23.6) 46 (37.4) 22 (17.9) 15 (12.2)	0.376
Birth order of child, n (%) First Second Third Fourth or higher	100 (41.2) 68 (28.0) 42 (17.3) 33 (13.6)	47 (39.2) 37 (30.8) 23 (19.2) 13 (10.8)	53 (43.1) 31 (25.2) 19 (15.4) 20 (16.3)	0.437
Married parent, n (%)	211 (86.8)	101 (84.2)	110 (89.4)	0.225
Family history of childhood enuresis, n (%)	108 (44.4)	65 (54.2)	43 (35.0)	0.003
Diagnosis of SCD in infancy, n (%)	88 (36.2)	49 (40.8)	39 (31.7)	0.139
Hospitalisation last 12 months, n (%)	99 (40.7)	51 (42.5)	48 (39.0)	0.581
History of previous transfusion, n (%)	143 (58.8)	72 (60.0)	71 (57.7)	0.718

### Predictors of enuresis in children with sickle cell anaemia

Among children with SCA, two covariates were found to be predictive of enuresis. Pre-adolescents had an adjusted odds ratio of 2.73 (1.50–4.96) of having enuresis compared with adolescents. In addition, children with a family history of childhood enuresis had an adjusted odd ratio of 2.60 (1.41–4.78) of having enuresis compared with those with no family history of enuresis. In contrast, the sex of the child, birth order, socioeconomic class, and neither sickle cell-related descriptors such as age at diagnosis of SCA less than 12 months, prior blood transfusion nor hospitalisation in the last 12 months before the study were independent predictors of enuresis in children with SCA (Table 5).

**Table 5:** Independent predictors of enuresis in children with sickle cell anaemia.

Independent variables	Adjusted odd ratio (95% confidence interval)	P value
Male versus female	1.49 (0.83–2.68)	0.180
Pre-adolescent versus adolescent	2.73 (1.50–4.96)	0.001
Number of children per family Two versus one Three versus one Four versus one Five or more versus one	2.49 (0.66–9.41) 1.87 (0.48–7.26) 4.15 (0.89–19.44) 2.03 (0.34–12.02)	0.178 0.365 0.071 0.434
Birth order Second versus first Third versus first Fourth or more versus first	0.97 (0.47–2.02) 0.85 (0.34–2.15) 0.51 (0.15–1.69)	0.934 0.733 0.270
Socioeconomic status Low versus high Middle versus high	2.02 (0.75–5.46) 1.00 (0.38–2.63)	0.165 1.00
Other marital status versus married	2.02 (0.82–4.93)	0.125
Family history of enuresis versus no family history	2.60 (1.41–4.78)	0.002
Previous blood transfusion history versus none	0.77 (0.42–1.42)	0.400
Diagnosis of SCD in infancy versus diagnosis at older age	0.57 (0.31–1.06)	0.078
Hospitalisation in the past one year versus none	1.73 (0.92–3.27)	0.089

## Discussion

We found high prevalence of enuresis of 49.4% in children and adolescents with SCA, which was about 70% higher than in age and sex-matched controls. The strong association between sickle cell disease and enuresis has been consistently reported in many studies. In Congo and Nigeria, Mabiala Babela [4] and Akinyanju [3], respectively, documented enuresis rates of 41.6% and 50.9% in children with sickle cell disease and 16.4% and 21.1% in controls. Similarly, a study by Readett [2] among 8-year old Jamaican children found enuresis, defined as at least one night of wet per month, in 54.9% of children with SCA compared with 26% in children with normal haemoglobin genotype. Our study further highlights the higher rate of enuresis in children with sickle cell disease in developing countries. The prevalence of enuresis in the present study was at least 1.5 times higher than the rates of 20–39% in children with SCA in Europe and United States of America [6, 9]. This higher rate of enuresis in developing countries than developed countries has been established in the general population of children and suggests a role for large families and socioeconomic disadvantage as additional drivers of enuresis in children [15–17]. It is also likely that lower uptake of proven therapies of enuresis in developing countries, as shown in the present study, means fewer children get cured of enuresis than in developed countries.

Several studies have focussed on the basis for the association of sickle cell disease and enuresis with inconsistent results. Hyposthenuria-induced nocturnal polyuria arising from medullary infarction is thought to be the most plausible mechanism for enuresis in SCA [5, 18]. However, in a study by Readett [7], children with SCA and enuresis had similar maximum voided urine volume and urine osmolality after water deprivation compared with those without enuresis. Instead, the study by Readett [7] documented that children with SCA and enuresis had functionally smaller bladder than those without enuresis. Furthermore, Portocarrero [19] and Anele [20] observed that symptoms of overactive bladder were more common in children and adults with sickle cell disease. Another commonly stated reason for enuresis is the presence of sleep disordered breathing, which in the general population, is also associated with enuresis [21, 22]. Hyperplasia of lymphoid organs and changes in the jaw configuration in children with SCA are thought to predispose these children to higher rates of sleep disordered breathing. Overall, children with SCA tend to have two times the rate of sleep disordered breathing compared to age matched controls. In a study by Lehmann [6], both the presence and severity of enuresis were significantly associated with sleep disordered breathing assessed by polysomnography. However, adenotonsillectomy is not routinely recommended for the treatment of enuresis because the results of studies in the general population have been conflicting and no such studies have been undertaken in children with SCA [23, 24].

Our findings highlight the fact that enuresis in children and adolescents with SCA is substantially different than in the general population. First, the well-reported decline in the prevalence of enuresis with age was less pronounced in children with SCA. In the present study, although enuresis occurred in 1.40 times more children aged 5–7 years with SCA than age and sex-matched controls, it occurred 2.38 and 5.0 times more often in children with SCA aged 11–13 years and 14–17 years than in their counterparts without SCA, indicating lower spontaneous resolution rates in those with SCA. The studies by Field [9]and Portocarrero [19] also documented persistently high rates of enuresis of 18% and 21% among late adolescents with SCA in USA and Brazil, respectively, although no comparison was made in adolescents without sickle cell disease in these studies. Second, children with SCA in the present study had two features of enuresis that further indicated a relatively difficult to treat type of enuresis: they were more likely to have more frequent wet nights per week and daytime symptom of urgency than the controls. Although, most studies have shown the high frequency of enuresis in children with sickle cell anaemia, few studies have commented on the frequency of wet nights per week and non-monosymptomatic enuresis. Lehmann [6] found severe enuresis, defined as at least three wet episodes per week, in 23.6% of unselected children with SCA, similar to the 22.2% in the present study. In Congo, Mabiala Babela [4] documented higher wet nights per week in children with sickle cell disease compared with those with normal haemoglobin genotype. In the present study, we documented non-monosymptomatic enuresis in about 60% of the children with SCA and enuresis, which was twice the percentage in the controls. Our finding corroborated the report of Portocarrero [19] which observed non-monosymptomatic enuresis in 58% of the children with sickle cell disease and enuresis. Although, these features are associated with poor outcome with therapy in the general population, one small study showed partial or complete resolution of enuresis in 60% of children with sickle cell disease who received desmopressin [15, 25]. In the absence of large studies comparing the response rate to desmopressin and alarm therapy in children with SCA and the general population, response to standard treatment in children with sickle cell and enuresis is unknown.

Similar to the studies by Ekinci [26] and Eneh [10], we found enuresis to be more common in children with a history of childhood enuresis in a parent or sibling, underlining a role for genetics and family dynamics in the aetiology of enuresis. This pattern of association is frequently reported in the general population [16, 27]. Furthermore, young age was also a strong predictor of enuresis in children with SCA in the present study, reflecting the differential maturation of the micturition centre. However, unlike in the general population, gender was not associated with enuresis in the present study. Whereas enuresis is consistently reported as more common in boys than girls in the general population [12, 16], its association with gender in children with sickle cell disease is less consistent. In the study by Akinyanju [3], enuresis prevalence was similar in boys and girls with SCA. In contrast, Mabiala Babela [4] reported higher enuresis rates in girls than in boys in Congo [4]. Furthermore, it is plausible that some sickle cell-related factors may be associated with enuresis. Readett [2] showed higher enuresis rate in children with SCA than in those with haemoglobin SC and lower fetal haemoglobin level in boys with sickle cell disease and enuresis but not in girls. Furthermore, Mabiala Babela [4] and Ekinci [26] documented higher hospitalisation and painful crisis rates in children with SCA with enuresis than in those without enuresis, respectively. In contrast, Field [9] reported no association between enuresis rate and severity markers of sickle cell disease such as acute chest syndrome and pain crisis [9]. Similar to the study of Field, we did not find any association between enuresis and hospitalisation in the 12 months before the study, receipt of blood transfusion and early diagnosis of SCA in the present study.

In spite of regular contact with the health system and high level of concern shown by the parents and caregivers in the study, only about 10% of the parents of children with SCA had spoken with a medical doctor about enuresis. This is much in form with the observation of many investigators, especially in developing countries, that parents keep their concern about enuresis in their children to themselves [16, 27]. In some cases, they employ measures to treat enuresis that may constitute child abuse such as physical punishment [28]. Therefore, we recommend that healthcare practitioners should regularly ask about enuresis in children with SCA and offer affected children referral to enuresis clinics.

## Limitations

We included about 40% of the control from a community study in which the haemoglobin genotype of the participants was unknown. However, we have no reason to think that the prevalence of SCA in the community would be more than the nationwide prevalence of 2–3% in Nigeria [29]. Having a control group similar in age, gender and proportion with family history of childhood enuresis helped mitigate the confounding effects of these factors on childhood enuresis. We also could not comment on urine frequency and presence of constipation in the study participants because we did not administer a voiding frequency diary and parents tend to overestimate or are unaware of the daytime bladder and bowel frequency especially of adolescents.

## Conclusion

We found very high rate of enuresis in children with SCA which persisted into late adolescence. Our study indicated that children with sickle cell anaemia had enuresis that was substantially different from the general population. They were older, more likely to have frequent wet nights per week and associated daytime symptoms of urgency than children without SCA. We identified young age and family history of childhood enuresis in a parent or sibling as predictors of enuresis in children with SCA. Despite the high rates of enuresis including more frequent wet nights and high level of parent concern, parents of children with SCA and enuresis, like parents of the controls, had not consulted a medical doctor about enuresis in their children. We recommend that healthcare practitioners discuss the issue of enuresis with parents of children with SCA. There is also a need to determine the most effective and affordable enuresis treatment modalities in children with SCA in developing countries.

## Supporting information

S1 Survey Instrument Survey questionnaire. (DOCX)

S1 Database Database for the study. (SAV)
