# Supplementary material for: Enuresis in children and adolescents with sickle cell anaemia is more frequent and substantially different from the general population
Source: PLoS One. 2018 Aug 10;13(8):e0201860. doi: 10.1371/journal.pone.0201860 (PMC6086414; doi:10.1371/journal.pone.0201860)
Supplement: S1 Survey Instrument — (DOCX) [file pone.0201860.s001.docx]

**Sickle Cell Disease and Urinary Incontinence: A Clinic-Based Study**

**A: To be Completed for All**

1. **Does your child wet his or herself with urine**: Yes [ ] or No [ ] **If the response *to Question 1*  is ‘No’, *Please skip to the next page***
2. **When does the wetting occurs**: When asleep **ONLY** **[ ]** When awake **ONLY** **[ ]** Either when **asleep or awake** **[ ]**
3. **How long has this bed wetting lasted:** Less than 3 months **[ ]** More than 3 months **[ ]**
4. **How often does your child wet his bed**: Once or twice/month **[ ]** 1 times/wk **[ ]** 2 times/wk **[ ]** 3 times/wk **[ ]**  4 times/wk **[ ]** 5 times/wk **[ ]** 6 times/wk **[ ]** 7 times/week **[ ]**
5. What is the **longest time** your child has been **dry *before wetting first* started:** Never **[ ]** Less than 6 months **[ ]** 6 months or more **[ ]**

**B: Section on Bed wetting.** To be completed **by ALL**

1. **Relationship of the respondent to the study participant (child)**: Mother **[ ]** Father **[ ]** Grandparent **[ ]** Others **[ ]**; pls specify:__________________________
2. **Respondent’s marital status**: Single **[ ]** Married **[ ]** Divorced **[ ]** Widowed/widowered **[ ]**
3. **Respondent’s highest educational level**: None **[ ]** Did not complete primary **[ ]** Completed primary **[ ]** Did not complete secondary **[ ]** ?

Completed secondary **[ ]** Tertiary **[ ]**, pls specify:_____________________________________

1. **Respondent’s spouse’s highest educational level**: None **[ ]** Did not complete primary **[ ]** Completed primary **[ ]** Did not complete secondary **[ ]** Completed secondary **[ ]** Tertiary **[ ],** pls specify:_______________________________________
2. **Respondent’s occupation**: be specific:_____________________________________ **Respondent’s spouse’s occupation: be specific**:___________________________
3. **The family occupies how many rooms (circle one)**: 1 2 3 4 5 6 7 8
4. **Study ID**:______/______**Date of Birth**:____/____/_____(*dd/mm/yy*) **Age of child:______** (years) **Gender of child**: M **[ ]** F **[ ]** **Birth order of index child**:______
5. **Number of children in the Family**:_________ **Present School level of child**: Primary **[ ]** Secondary **[ ]** Tertiary **[ ]**
6. **What is the present school class of the child** (*please circle one*) : 1 2 3 4 5 6 **Is child’s school a boarding facility:** Yes **[ ]** No **[ ]**
7. **Contact Phone number:___________________________/____________________________ Hb Genotype: SS [ ] SC [ ] AS [ ] AA [ ] Unknown [ ]**
8. **Initials of Interviewer**:­­­­­­­­­­­­­­­­______**Date of Interview**: _____/_____/______(dd/mm/yy) **Respondent’s Tribe**:________ **Respondent’s Religion: ______________________**
9. In the past one month which of the following does the child have **during the day**? **Tick the appropriate respon**
10. **How often does the child pass stools**: 1-2 times/day **[ ]** Once in 2-3 days **[ ]** Once in 4-5 days **[ ]** Once in a week **[ ]** Others **[ ]**
11. **Most often the stools passed by the child are**: Soft/paste like **[ ]** Hard **[ ]** Pellet-like **[ ]**
12. *More often than not* **is passing stools painful** : Yes **[ ]** or No **[ ]** *Most nights****,* does the child snore heavily**: Yes **[ ]** or No **[ ]**
13. **Does any of child’s sibling (s) bed wets or had urine wetting problem in the past:** Yes **[ ]** No **[ ]** **Did any of the parents bed wet as a child:** Yes **[ ]** No **[ ]**

**C.** To be completed by **parent of child who bed wet ONLY**

1. **On a scale of 1 to 10 (1 means ‘no effect’, while 10 means ‘greatly affected’), how do you think bedwetting has affected the child (circle one option)**: 1 2 3 4 5 6 7 8 9 10
2. **On a scale of 1 to 10 (1 means ‘not concerned’ while 10 means ‘greatly concerned’), how concerned are you about your child’s wetting problem (circle one option):** 1 2 3 4 5 6 7 8 9 10
3. **Have you ever spoken to a doctor about your child’s wetting problem**: Yes **[ ]** or No **[ ]**
4. **What intervention has been used to treat bedwetting in the child (can choose more than one options):** Does nothing about it **[ ]** Punishes the child**[ ]** Wakes up the child every night to urinate **[ ]** Gives child herbal medications **[ ]** Uses alarm clock **[ ]** Uses prescribed medications **[ ]** Restricts fluid intake in the evening

**[ ]** Others **[ ],** please specify:_____________________________________

1. At what age was the child diagnosed to have sickle cell disease: ------------ (years)
2. How many hospital admissions in the **past 12 months for any illness** --------
3. How many admissions for painful crisis in the **past 12 months:** -----------------
4. How many units of blood did the child receive in the **past 12 months**:-----------
5. When was the last time the child received blood transfusion: ---------------

| **Complications** | **Current** | **Previous** |
| --- | --- | --- |
| Leg ulcers |  |  |
| Acute chest syndrome/pneumonia |  |  |
| Avascular necrosis |  |  |
| Stroke |  |  |
| Priapism (males only) |  |  |
| Kidney disease |  |  |

1. **Characteristics of Sickle Cell Disease (For those with SICKLE CELL ONLY)**

Tick which of the following complications of SCD the child has/had:

| **Complications** | **Current** | **Previous** |
| --- | --- | --- |
| Leg ulcers |  |  |
| Acute chest syndrome/pneumonia |  |  |
| Avascular necrosis |  |  |
| Stroke |  |  |
| Priapism (males only) |  |  |
| Kidney disease |  |  |

| Questions | Yes | No | Questions | Yes | No |
| --- | --- | --- | --- | --- | --- |
| 1. Have underpants that are wet with urine |  |  | 1. Cannot hold urine for a while even when told to do so |  |  |
| 1. Strains when passing urine |  |  | 1. Runs rather than walk to the toilet to prevent wetting |  |  |
| 1. Squats or uses other techniques to prevent wetting |  |  |  |  |  |
